# Use of a Multiplex Immunoassay Platform to Investigate Multifaceted Antibody Responses in SARS-CoV-2 Vaccinees with and Without Prior Infection

**DOI:** 10.3390/covid5040044

**Published:** 2025-03-22

**Authors:** Troy Odo, Brien K. Haun, Caitlin A. Williams, Aquena Ball, Albert To, Teri Ann S. Wong, Lauren Ching, Eileen Nakano, Alex Van Ry, Laurent Pessaint, Hanne Andersen, Oreola Donini, Vivek R. Nerurkar, Axel T. Lehrer

**Affiliations:** 1 Department of Tropical Medicine, Medical Microbiology, and Pharmacology, University of Hawaii Manoa, Honolulu, HI 96813, USA; 2 Cell and Molecular Biology Graduate Program, University of Hawaii Manoa, Honolulu, HI 96813, USA; 3 Bioqual Inc., Rockville, MD 20850, USA; 4 Soligenix, Inc., Princeton, NJ 08540, USA

**Keywords:** SARS-CoV-2, subunit vaccine, mRNA vaccine, multiplex immunoassay, humoral immunity, multiplex inhibition test, avidity

## Abstract

The emergence of COVID-19 necessitated the rapid development of vaccines. While highly effective at reducing severe disease and death, breakthrough infections remain a problem as the virus continues to mutate. To help address this issue, we show the utility of a multiplex immunoassay in measuring multiple aspects of the antibody response generated by SARS-CoV-2 vaccines. We use a multiplex immunoassay platform to measure spike-specific IgG concentration, avidity, and receptor-binding inhibition. In addition, we correlate results from an ACE-2 receptor-binding inhibition assay with corresponding data from a SARS-CoV-2 microneutralization assay to establish this inhibitory assay as a potential predictor of virus neutralization. We studied these antibody responses in SARS-CoV-2-naïve and -convalescent vaccinees. Our results showed increased IgG concentrations, avidity, and inhibition following vaccination in both groups. We were also able to differentiate the immune response between the two groups using the multiplex immunoassay platform to look at antibody diversity. The receptor-binding inhibition assay has strong correlations with a cell-based pseudovirus neutralization assay as well as with WT SARS-CoV-2 Washington and Delta variant PRNT_50_ assays. This suggests that the inhibition assay may be able to simultaneously predict virus neutralization of different SARS-CoV-2 variants. Overall, we show that the developed custom multiplex immunoassay with several experimental variations is a powerful tool in assessing multiple aspects of the SARS-CoV-2 antibody response in vaccinated individuals.

## 1. Introduction

An outbreak of viral pneumonia was first identified at the end of 2019 in Wuhan, China. The etiological agent was quickly identified as a novel coronavirus originally named 2019 novel coronavirus (2019-nCoV). The name was later changed to severe acute respiratory virus coronavirus 2 (SARS-CoV-2). Coronavirus disease 2019 (COVID-19) quickly spread throughout the world, leading the World Health Organization (WHO) to declare a global pandemic in March 2020. An unprecedented effort led to the rapid development of multiple SARS-CoV-2 vaccines which were shown to be both safe and effective in helping to prevent infection and hospitalizations [1]. However, despite the historic success of the vaccination campaigns, breakthrough infections remain an issue due to waning vaccine efficacy as well as antigenic variation in new and emerging variants [2–4].

Antiviral immunity requires a multifaceted response which incorporates multiple aspects of the immune system. The innate immune response and the cellular adaptive immune response are important for controlling disease following infection [5–7]. However, humoral immunity, specifically antibody responses, have been well established as having a major role in vaccine immunity and disease prevention [8–10]. There are many aspects of antibody-mediated immunity that may help to confer protection against disease and infection, including antibody concentration, virus-neutralizing antibody titers, and antigen–antibody-binding strength. Antibody concentration and neutralizing antibody titers are mostly associated with a protective immune response, and therefore, SARS-CoV-2 vaccine research focused heavily on these two measurements [11–13]. Additionally, there is evidence suggesting that antibody avidity may also play an important role in antiviral immunity [14–16].

Virus neutralization is an important effector function of antibodies. The potential role of virus-neutralizing antibodies in SARS-CoV-2 immunity has been extensively studied, and while it is thought to play a major role in protection, it has not been established as a correlate of protection against SARS-CoV-2 [17]. In general, neutralizing antibodies rely on blocking a single interaction between its target antigen and cognate receptor which results in a highly specific response [18–20]. This also limits the breadth of response, making it difficult to generate cross-reactive neutralizing antibodies. There may also be an important relationship between antibody avidity and virus neutralization. The SARS-CoV-2 spike protein and human ACE2 bind with high affinity as is typical for a virus–receptor interaction; therefore, neutralizing antibodies should have high avidity for the spike protein to reliably outcompete the high affinity binding of the spike protein and ACE2 facilitating cell entry [21]. Studying avidity could provide a measure of affinity maturation, an important process in the development of an effective antibody response as well as a lasting memory response. Studies have also shown that there may be differences in avidity maturation induced by natural infection and vaccination [22–24]. Vaccinated individuals in these studies developed higher avidity titers than convalescent (previously infected) individuals, but only after receiving two or more vaccine doses. Additionally, the results suggest that the development of high-avidity antibodies is important for cross-protection and limiting breakthrough infection.

There are a variety of immunoassays that can be used for antibody detection, both clinically and in research settings. For SARS-CoV-2 particularly, ELISAs, Western blots, lateral flow assays, and multiplex bead-based assays have all been used to detect IgG, IgM, and IgA responses to multiple viral proteins [25–27]. Multiplex assays are of particular interest because of the unique advantages they hold over conventional immunoassays, including improved sensitivity and specificity, due to a greater dynamic range compared to assays such as ELISAs [28,29]. Additionally, multiplex assays can reduce time and resources since different antigen-specific antibodies can simultaneously be detected by multiple antigens coupled to microspheres with unique fluorescent signatures.

While traditional virus neutralization assays using relevant isolates of SARS-CoV-2 are valued for their sensitivity and specificity, one of the drawbacks is that they are resource-intensive and require specialized BSL-3 testing facilities. Researchers also use pseudovirus neutralization assays utilizing attenuated, recombinant viral strains that are pseudotyped with the target virus’ surface protein. While this method does eliminate the need for BSL-3 facilities, there are still stringent cold-chain requirements for virus storage, which may not be available in all parts of the world. To address these issues, we developed a multiplex immunoassay (MIA)-based multiplex inhibition test (MINT) that could serve as a safe and convenient alternative to studying virus neutralization [30].

Here, we describe the utility of a multiplex bead-based platform to measure multiple facets of the antibody response to vaccination in SARS-CoV-2-naïve and -convalescent individuals. To conduct this, we used the multiplex assay to observe longitudinal changes in antigen-specific IgG concentration, antibody avidity, and receptor-binding domain inhibition in both the naïve and convalescent populations. We show that the multiplex immunoassay platform can differentiate between antibody responses in the two populations. Additionally, we demonstrate that receptor-binding inhibition correlates with virus neutralization for multiple SARS-CoV-2 variants, including in a non-human primate vaccine booster study. For this study, we follow the early evolution of the virus and focus primarily on the Wuhan (Wuhan-Hu-1/2019) and Washington (USA-WA1/2020) isolates (which are equivalent [31] and will be referred to as the “prototype” strain) and the Delta (B.1.617) variant.

## 2. Materials and Methods

### 2.1. Human Serum Samples

Longitudinal samples were collected from individuals who did (convalescent) or did not (naïve) have a history of a prior SARS-CoV-2 infection. All participants were vaccinated with mRNA vaccines. For naïve individuals, serum samples were collected prior to vaccination (pre), 14–28 days post-dose 1 (PD1), and 14–28 days post-dose 2 (PD2). Convalescent serum samples were collected from individuals who self-reported SARS-CoV-2 infection through positive results from PCR testing. Serum samples for all individuals in the convalescent group were collected prior to vaccination, but after SARS-CoV-2 infection (between 96 days and 354 days after PCR-positive testing), 14–28 days PD1, and 14–28 days PD2.

### 2.2. Antibody Concentrations

Antibody concentrations in human sera were measured using a multiplexed immunoassay. Magplex^®^ beads (Luminex, Austin, TX, USA) were coupled to pre-fusion stabilized, full-length SARS-CoV-2 spike protein. A master mix containing full-length spike and BSA-coupled beads as a negative control was diluted at 1:200 with MIA buffer (1× PBS + 1% BSA + 0.02% Tween 20). Serum samples were serially diluted two-fold from 1:500 to 1:32,000 with MIA buffer. A polyclonal standard of anti-SARS-CoV-2 Spike human IgG was used to determine IgG concentrations by interpolation on a standard curve. A polyclonal IgG standard was purified by two-step affinity chromatography using Protein A followed by an NHS-Sepharose column coupled covalently with recombinant SARS-CoV-2 spike protein [25]. The standard was serially diluted 2-fold from 4000 ng/mL to 1.95 ng/mL. A total of 50 μL of serum dilutions were plated in duplicate wells on a 96-well plate. A total of 50 μL of bead master mix was added to each well, and the bead–serum mixture was incubated at 37 °C for 3 h while shaking at 700 rpm. After incubation, the plates were placed on a plate magnet for 2 min, and the supernatants were removed. The beads were then washed twice with 200 μL of MIA buffer. (R-PE)-conjugated goat anti-human IgG antibody (1 ug/uL) was diluted with MIA buffer at 1:200, and 50 μL of the secondary antibody dilution was added to each well. Plates were incubated for 1 h at 37 °C on a shaker at 700 rpm. Following incubation, the beads were washed twice, and finally, 120 μL of MAGPIX^®^ drive fluid was added to the beads in each well. Plates were read on a MAGPIX^®^ instrument (Luminex Corporation, Austin, TX, USA). A standard curve established using the IgG standard was run with each assay and used to determine serum anti-spike IgG concentrations by interpolation using Prism (GraphPad Software, Version 10.2.3, Boston, MA, USA).

### 2.3. Antibody Avidity Assay

Antibody avidity was determined using a chaotrope-modified version of the MIA previously described [32]. Human serum was diluted to a concentration of approximately 80 ng/mL, based on previously determined IgG concentrations, to achieve similar IgG saturation on the assay beads. A total of 50 μL of these dilutions were incubated with antigen-coupled microsphere beads for 3 h at 37 °C. Incubation was followed by two washes with MIA buffer. Duplicate wells of antibody–bead complexes were then incubated with 50 μL of increasing concentrations (0.5 M, 1 M, 2 M, 4 M, or 6 M) of ammonium thiocyanate (NH_4_SCN) for 10 min at 37 °C on a shaker at 700 rpm. MIA buffer was added to the control wells that were not treated with NH_4_SCN. Three rounds of washing with MIA buffer were then followed by a one-hour incubation with (R-PE)-conjugated goat anti-human IgG antibodies as in the standard MIA procedure. After washing with the MIA buffer twice, the beads were resuspended in MAGPIX^®^ drive fluid and analyzed on a MAGPIX^®^ instrument as described above. Relative avidity was calculated at each concentration of NH_4_SCN by dividing the median fluorescence intensity (MFI) measured at each chaotrope concentration by the MFI of the MIA buffer-treated negative control of that same sample. For example, the 4 M relative avidity of a sample was calculated by dividing the MFI of a sample following 4 M NH_4_SCN treatment by the MFI of the PBS treatment of that sample. The chaotrope concentration at which 50% MFI remained was interpolated using Prism and reported as the avidity titer 50% cut-off (AT_50_).

### 2.4. Multiplex Inhibition Test (MINT)

Receptor-binding inhibition was determined using a modified version of the MIA [30]. In brief, human sera were serially diluted four-fold from 1:25 to 1:25,600 and incubated with antigen-coupled microspheres for 3 h at 37 °C. Incubation was followed by two washes with MIA buffer. A total of 200 ng of R-PE-conjugated hACE-2, produced as described previously [30], was added in 50 μL of buffer to each well of the 96-well plate and incubated for 2 h. After washing with the MIA buffer twice, the beads were resuspended in MAGPIX^®^ drive fluid and analyzed on a MAGPIX^®^ instrument. A binding curve was generated based on the serum dilutions, and the serum dilution at which 50% inhibition occurred (MINT_50_) was calculated using non-linear regression analysis in Prism.

### 2.5. Pseudovirus Microneutralization Assay

Virus-neutralizing titers of the serum samples were determined with a microneutralization test. rVSVΔG-SARS-CoV-2-GFP, containing the spike protein of the prototype variant, obtained from Rocky Mountain Laboratories (Hamilton, MT, USA), was propagated in Vero cells. One day before the assay (day 0), Vero cells were seeded in a 96-well plate at 2.5 × 10^4^ cells per well in 100 μL of Dulbecco’s Modified Eagle Medium supplemented with 10% Fetal Bovine Serum (DMEM-10) (Gibco, Waltham, MA, USA). On day 1, serum samples were heat-inactivated at 56 °C for 30 min before use. Sera were diluted four-fold from 1:40 to 1:40,960 and incubated with 2 × 10^4^ PFUs/mL of rVSV-SARS-CoV-2-GFP in DMEM-2 medium (125 μL) for 1 h. Following incubation, the serum and virus mixtures were added to Vero cells in quadruplicate and incubated for 22 h. Following incubation, cells were fixed with 40 μL of 1% paraformaldehyde and subsequently washed 3 times with PBS. The plates were then visualized and counted using the CTL Immunospot reader S6 (Cellular Technology Limited, Cleveland, OH, USA). The serum dilution at which 50% virus inhibition occurred (NT_50_) was reported as the virus neutralization titer. The NT_50_ titers were determined by variable-slope non-linear regression analysis in Prism. The lowest serum dilution, 1:40, defined the negative cut-off. Negative values are represented as an NT_50_ of 20 in all figures to allow for statistical analysis.

### 2.6. Non-Human Primate (NHP) Study Design

Cynomolgus macaques (n = 5) were initially immunized via the IM route with 2 doses of AZD1222 (formerly ChAdOx1 nCoV-19) SARS-CoV-2 vaccine 4 weeks apart (not part of the current study). Thirty-five weeks later, the animals received a recombinant protein subunit vaccine booster (25 ug SARS-CoV-2 S protein + 5 mg CoVaccine HT^™^) as previously described [25]. NHPs were immunized intramuscularly (IM) in both deltoids (split dose). Serum samples were collected at baseline (1 week prior to booster administration), 3 days after booster administration, and at weeks 1, 3, and 13 following the booster. Serum samples were immediately processed, aliquoted, and stored at −80 °C until analysis.

### 2.7. Recombinant Protein Expression and Purification

The plasmid pHH202 (Hawaii Biotech Inc., Honolulu, HI, USA) was generated to express the pre-fusion, protease-resistant, trimeric transmembrane-deleted SARS-CoV-2 spike (S) glycoprotein as previously described [33]. Following transfection into Drosophila S2 cells using Lipofectamine LTX with PLUS reagent (Invitrogen, Carlsbad, CA, USA) stable transfected cell lines were selected using 300 μg/mL of hygromycin B. Stably transformed cultures were induced with culture medium containing 200 μM CuSO_4_. Protein expression was verified using sodium dodecyl sulfate polyacrylamide gel electrophoresis (SDS-PAGE) and Western blotting.

Recombinant S protein was purified from clarified cell culture supernatants by immunoaffinity chromatography (IAC) using the SARS-CoV-2 cross-reactive mAb CR3022 (obtained from Mapp Biopharmaceutical, San Diego, CA, USA, RRID:AB_2848080) coupled to NHS-activated Sepharose (Cytiva, Marlborough, MA, USA) at a concentration of 10 mg/mL. The antigen was eluted with a glycine buffer (pH 2.0) onto a HiPrep 26/10 desalting column (Cytiva, Marlborough, MA, USA) for immediate buffer exchange into PBS. Antigens were concentrated using Amicon filtration devices (EMD Millipore, Billerica, MA), filter-sterilized with a 0.22 μm syringe filter (Cytiva, Marlborough, MA, USA), and stored at −80 °C until use.

### 2.8. Wild-Type SARS-CoV-2 Virus Assay

Virus neutralization was determined using a plaque reduction neutralization test (PRNT). Sera from NHPs were initially diluted 1/10; this was followed by 3-fold serial dilutions. Diluted sera were incubated with 30 plaque-forming units (PFUs) of wild-type SARS-CoV-2 prototype (USA-WA1/2020; BEI-NR=52281) or Delta (B.1.617; BEI NR-55282) variants in equal volumes of culture medium for 1 h at 37 °C. Following incubation, serum–virus mixtures were added to a monolayer of confluent Vero E6 cells and incubated for 1 h at 37 °C in 5% CO_2_. Each well was overlaid with culture medium containing 0.5% methylcellulose and incubated for 3 days at 37 °C in 5% CO_2_. After 3 days, the plates were fixed with methanol at −20 °C for 30 min and then stained with 0.2% crystal violet for 30 min at room temperature. PRNT_50_ titers were calculated using variable-slope non-linear regression analysis in Prism.

## 3. Results

### 3.1. Anti-Spike Antibody Concentrations

**Figure 1.**
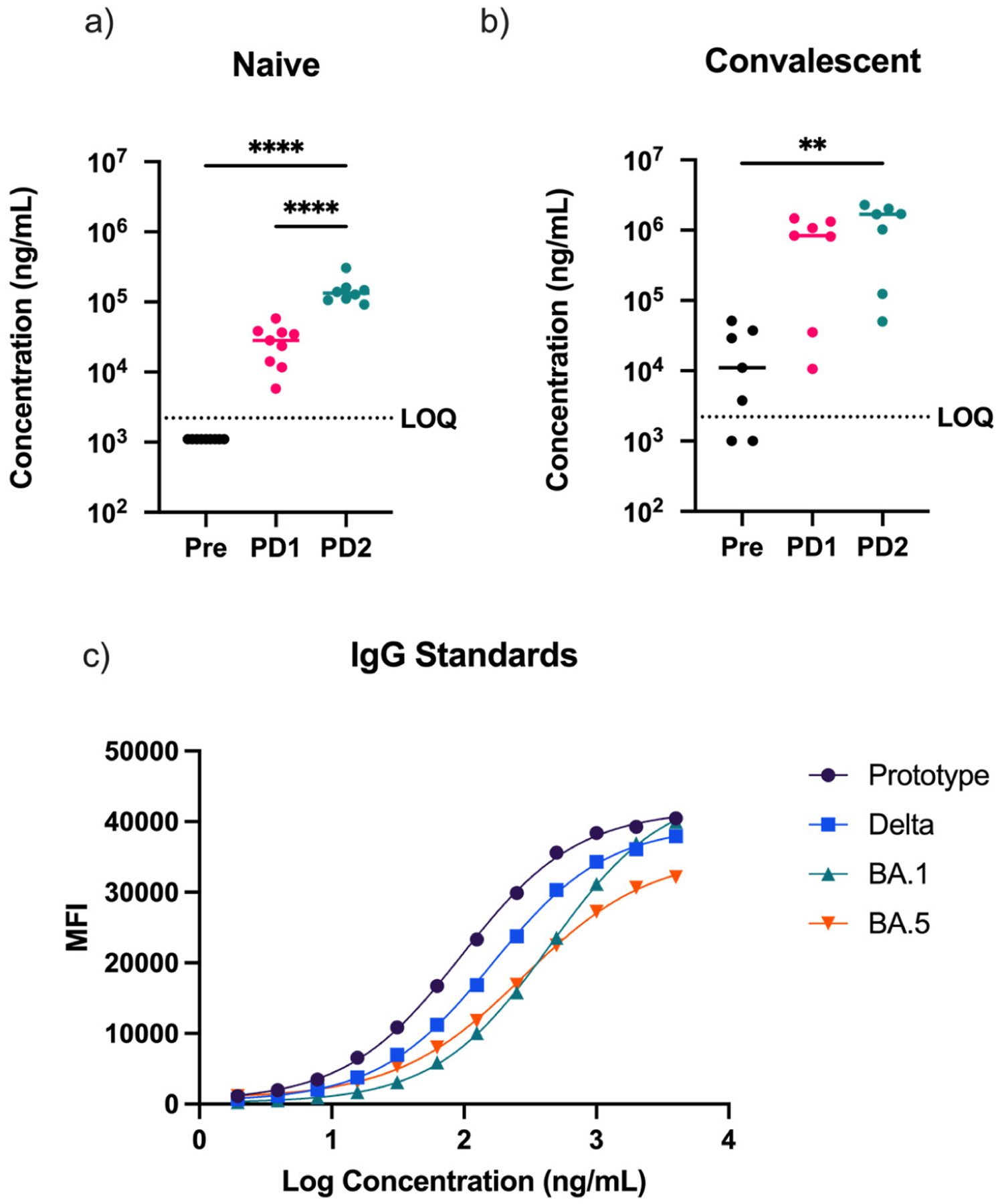
Longitudinal changes in anti-spike antibody concentrations in naïve and convalescent vaccinees. Anti-spike antibody concentrations were measured prior to vaccination (pre), >14 days post-dose 1 (PD1), and >14 days post-dose 2 (PD2) in SARS-CoV-2-naïve (**a**) and -convalescent (**b**) individuals (LOQ: limit of quantification). (**c**) Serum antibody concentrations were interpolated using purified SARS-CoV-2 prototype-specific spike protein. The polyclonal standard was reacted with multiple spike variants in addition to the prototype spike to measure binding characteristics against the Delta, BA.1, and BA.5 variants (one-way ANOVA, ** *p* < 0.01, **** *p* < 0.0001).

Serum samples of individuals with no prior infection history (naïve) were collected between January and March of 2021. Naïve individuals were self-reported to have had no SARS-CoV-2 infection, meaning an absence of COVID-19 symptoms and no positive SARS-CoV-2 PCR test results prior to vaccination. It is known that SARS-CoV-2 can cause mild or even subclinical infection, and there is a possibility that individuals, especially during the beginning of the COVID-19 pandemic, may not have been aware that they were infected. Since COVID-19 history was self-reported by participants in this study, it was important to determine pre-vaccination serum anti-spike IgG levels in the naïve group to additionally confirm a serologically negative history of infection. Anti-spike IgG concentrations were interpolated from a polyclonal anti-spike IgG standard (Figure 1c). Anti-spike IgG was below the limit of detection for all naïve individuals (Figure 1a), confirming that individuals in this group were serologically SARS-CoV-2-naïve prior to receiving vaccination. As expected, following the first round of vaccination (with first-generation mRNA vaccine), all previously naïve individuals developed detectable levels of anti-spike IgG. Anti-spike IgG concentrations also increased significantly following the second dose of vaccine (Figure 1a).

Serum samples of individuals with prior SARS-CoV-2 infection history were collected between August 2020 and April 2021. Individuals in the convalescent group self-reported PCR-positive test results as confirmation of SARS-CoV-2 infection prior to receiving vaccination. Based on self-reported data, dates of infection ranged from March 2020 through September 2020. During this time, the original prototype variant and derivatives were the primary circulating variants. Serum anti-spike IgG concentrations were measured to confirm prior infection status. Interestingly, although five of the seven convalescent samples had detectable serum anti-spike IgG, two samples fell below the limit of quantification (Figure 1b). Of these two, one individual self-reported asymptomatic infection. The other individual had an interval of 258 days between infection and sample collection, which may have resulted in diminished serum IgG. It is also possible that, although both individuals experienced exposure to SARS-CoV-2 resulting in a PCR+ test, the antigen exposure may have been insufficient to induce an IgG response to the SARS-CoV-2 spike protein in these individuals. In convalescent individuals, anti-spike IgG concentrations increased approximately 100-fold following the first vaccination except for the two participants that serologically tested negative and whose increases followed those observed in the naïve individuals (Figure 1b). Following dose 2 in the convalescent group, there was a smaller, non-significant increase in anti-spike IgG concentration. When compared to the increase seen in antibody concentration following dose 2 in the naïve group (5-fold increase), the convalescent group saw a markedly smaller increase (1.5-fold increase). This could be due to antibody titers reaching near-maximum levels following dose 2 in the convalescent group or the possibility that the interval between dose 1 and dose 2 was too short to be able to notice a booster response in convalescent individuals with pre-existing humoral immunity.

**Figure 2.**
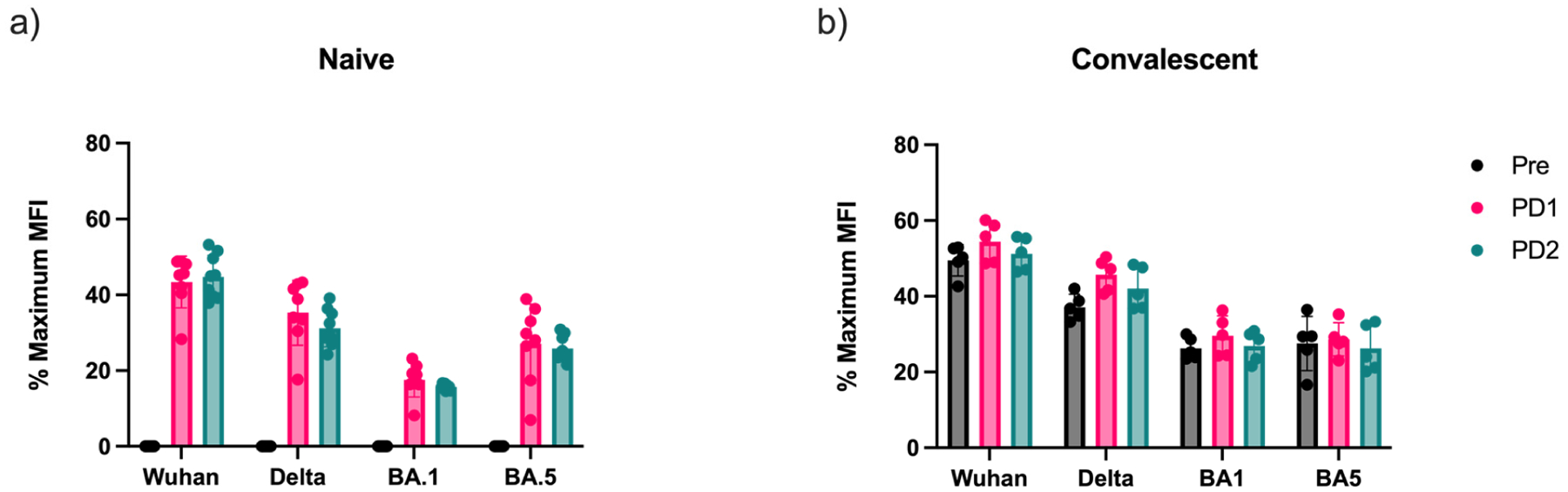
Antibody cross-reactivity towards multiple SARS-CoV-2 variants of concern in naïve and convalescent individuals. Antibody cross-reactivity was measured as the percentage of MFI of individual serum samples against each variant spike protein to the maximum MFI value of the standard curve for each of the different variants to normalize for different maximal MFI values achieved with different antigen-coupled beads. Anti-spike antibody concentrations were measured prior to vaccination (pre), >14 days post-dose 1 (PD1), and >14 days post-dose 2 (PD2) in SARS-CoV-2-naïve (**a**) and -convalescent (**b**) individuals.

To further expand on our analysis of SARS-CoV-2-specific antibody concentration, we wanted to examine antibody cross-reactivity to different SARS-CoV-2 variants. The polyclonal IgG standard was generated specifically for the prototype variant, as the vaccinated donors were immunized with the first-generation mRNA vaccines, preventing us from directly comparing IgG concentrations against different variants. To address this issue, we standardized readouts by determining the percent MFI of each sample relative to the maximum MFI reading observed with one of our IgG standards against each of the variant spike proteins. For example, when run against our SARS-CoV-2 spike protein bead panel (which included the prototype, Delta, as well as Omicron BA.1 and BA.5 spike proteins), our polyclonal IgG standard generated four different standard curves (see Figure 1c). We calculated the percent reactivity based on the MFI of each sample for the different antigens relative to the maximum MFI of the respective standard curve. In the naïve group, anti-spike IgG reactivity against the prototype variant increased following each vaccine dose (Figure 2a). However, this trend did not hold for the other variants tested. Relative IgG reactivity towards each of the other variants decreased following vaccination. This suggests that the mRNA vaccines administered to study participants elicited a skewed response, increasing specificity to the prototype variant but not cross-reactive antibody titers against other variants of concern. In the convalescent group, we saw an increase in reactivity following dose 1 for all variants (Figure 2b). Anti-prototype and Delta variant IgG showed the largest increase in reactivity, but we also saw small increases in reactivity to Omicron BA.1 and BA.5 as well. Interestingly, post-dose 2, the reactivity decreased for each variant. Percent reactivity was highest for the prototype, followed by the Delta and then the Omicron variants, as expected based on the antigenic relatedness of the variants and the timing of sample collection well prior to the emergence of the Delta variant. However, the increase in reactivity to Delta and BA.1 following dose 1 in the convalescent group, which was not seen in the naïve group, suggests that some cross-reactive immune responses were induced by natural infection and then subsequently boosted by mRNA vaccines based on the original prototype SARS-CoV-2 strains.

### 3.2. Antibody Avidity

In addition to measuring total antigen-specific antibody concentration, it is also important to analyze the quality of the antibodies produced through vaccination and natural infection. One measure of antibody quality is avidity. Antibody avidity, or the total binding strength of all interactions between antibodies and antigens, provides an indirect measure of affinity maturation, an important step in the development of the humoral immune response. To measure avidity, we used a chaotrope-modified version of the MIA previously used to measure antibody concentration. Increasing concentrations of chaotrope were added, and the relative avidity index, or the percentage of bound antibody, was calculated for each concentration.

**Figure 3.**
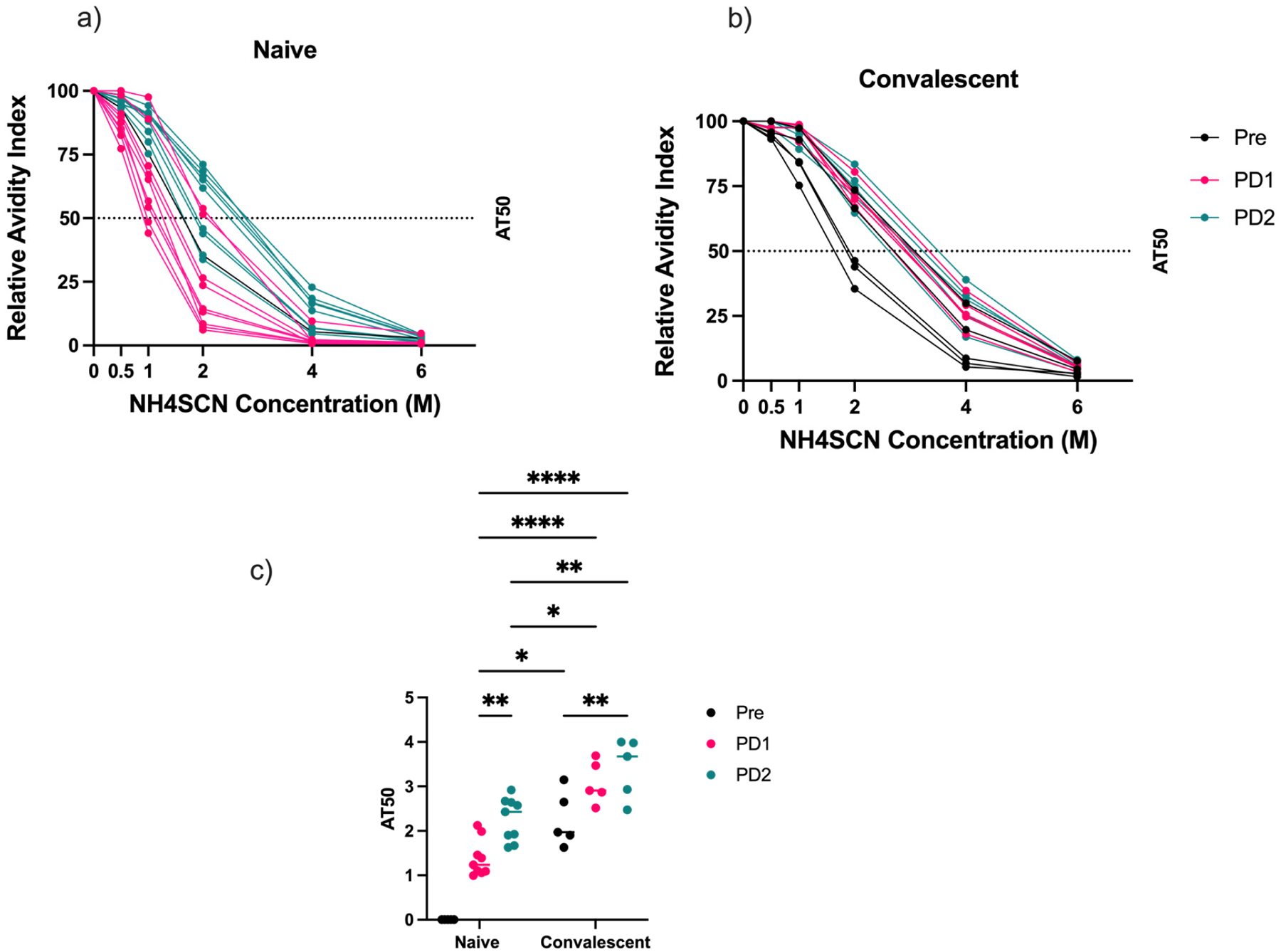
Changes in antibody avidity following vaccination in SARS-CoV-2-naïve and -convalescent vaccinees. Antibody avidity was measured using the relative avidity index in (**a**) naïve and (**b**) convalescent individuals. The relative avidity index was calculated as the percentage of bound antibody at different concentrations of chaotrope relative to a no-chaotrope treatment control. (**c**) The chaotrope concentration that dissociated 50% of antibody–antigen interactions (AT_50_) was determined and plotted for individual samples to compare relative avidity between groups at different timepoints (two-way ANOVA, * *p* < 0.05, ** *p* < 0.01, **** *p* < 0.0001).

The relative avidity index was calculated for pre-vaccination, PD1, and PD2 samples for both naïve (Figure 3a) and convalescent (Figure 3b) individuals. A non-linear regression curve was plotted for each group, and the concentration of NH_4_SCN that removed 50 percent of antibody (AT_50_) was determined. The two individuals with low pre-vaccination IgG concentrations were excluded from these data as they did not show the characteristics of previously exposed individuals. In the naïve group, avidity increased following both the first and second vaccine doses (Figure 3c), as expected. In the convalescent group, avidity increased after dose 1 and dose 2, albeit not significantly. However, even after a single dose in the convalescent group, avidity titers were higher than in naïve individuals who received two vaccine doses. In fact, some of the convalescent individuals who had not yet been vaccinated showed higher AT_50_ titers than naïve individuals who were vaccinated twice (Figure 3c). These results suggest that vaccination stimulates immune maturation. In naïve individuals, two vaccination doses were necessary to stimulate a relatively high avidity response; however, it would be interesting to investigate the effect of additional antigen exposure on avidity, whether it be through additional vaccine boosts or breakthrough infection. The results from the convalescent group suggest that hybrid immunity consisting of infection and vaccination generates a higher avidity response than two vaccine doses alone. The mechanisms behind these differences are unclear and should be investigated further.

### 3.3. Antibody-Mediated Receptor-Binding Inhibition

One of the hallmark measures of antibody function is virus neutralization. Virus neutralization is the process through which antibodies prevent viruses from infecting host cells. An essential step in this process is the inhibition of binding between viral surface proteins and host membrane proteins. In the case of SARS-CoV-2, the inhibition of binding between the SARS-CoV-2 spike protein and human ACE-2, its main cellular receptor, helps facilitate virus neutralization. Traditional virus neutralization assays are resource-intensive, time-consuming, and require specialized testing facilities. To address a few of these issues, we used a multiplex inhibition test (MINT) to measure virus inhibition using the same antigen-coupled beads as used for other MIA variations described here. This modified MIA incorporated incubation with a PE-labeled hACE-2 protein following antigen–sera incubation instead of using a fluorescently labeled secondary antibody. Samples were serially diluted, and the percent inhibition was calculated at each dilution.

**Figure 4.**
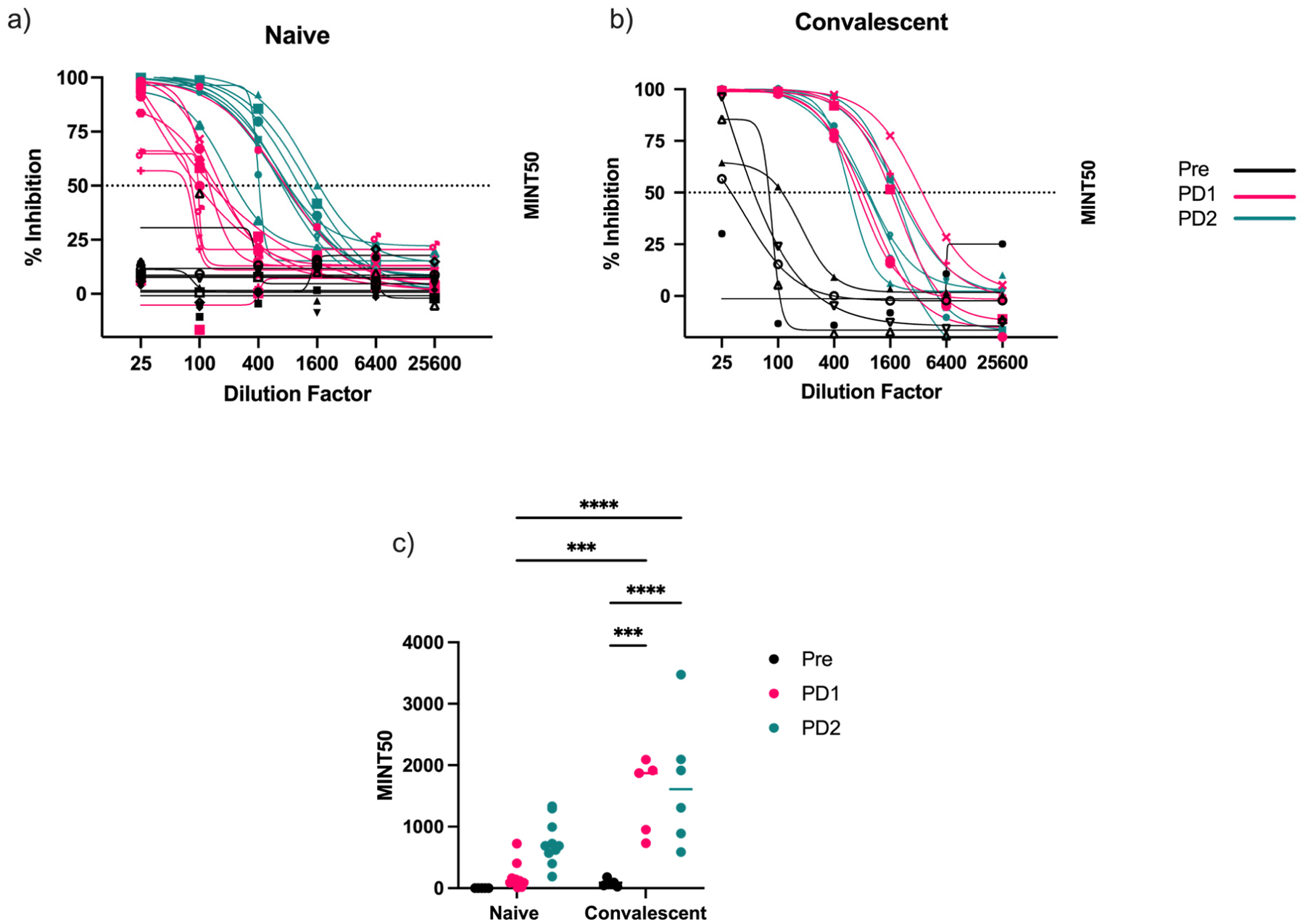
SARS-CoV-2 receptor-binding inhibition in naïve and convalescent vaccinees. Percent inhibition of ACE-2 binding was determined in serially diluted serum samples in (**a**) naïve and (**b**) convalescent individuals. Percent inhibition was calculated relative to negative control sample in which no inhibiting antibodies were present. (**c**) MINT_50_ values (dilution factor at which 50% hACE-2 binding was achieved) were calculated by interpolation to determine relative inhibition in naïve and convalescent sera (two-way ANOVA, *** *p* < 0.001, **** *p* < 0.0001).

In the naïve group, there was a clear increase in the inhibition of receptor binding following each vaccine dose as expected (Figure 4a), suggesting an increase in virus-neutralizing titers. Like antibody concentrations and avidity, the naïve group showed no inhibitory activity, providing further evidence of their SARS-CoV-2-naïve immune status. There was a similar trend in the convalescent group (Figure 4b), although the increase following dose 1 was greater than that following dose 2. To better clarify these results, we calculated the MINT_50_ value (the dilution factor at which 50% inhibition was achieved). There was a significant increase in inhibition following dose 1, but no further increase was observed following dose 2 (Figure 4c). Interestingly, the convalescent pre-vaccination group had low MINT_50_ titers that rapidly increased after a single vaccination. However, the results of the convalescent pre-vaccination samples appear in a similar range as compared to MINT_50_ titers for the naïve post-dose 1 group, indicating an absence of virus neutralization after natural infection. In both the IgG concentration and avidity assays, the convalescent pre-vaccination and naïve PD1 groups showed similar trends.

### 3.4. Virus-Neutralizing Antibodies Correlate with Receptor-Binding Inhibition

**Figure 5.**
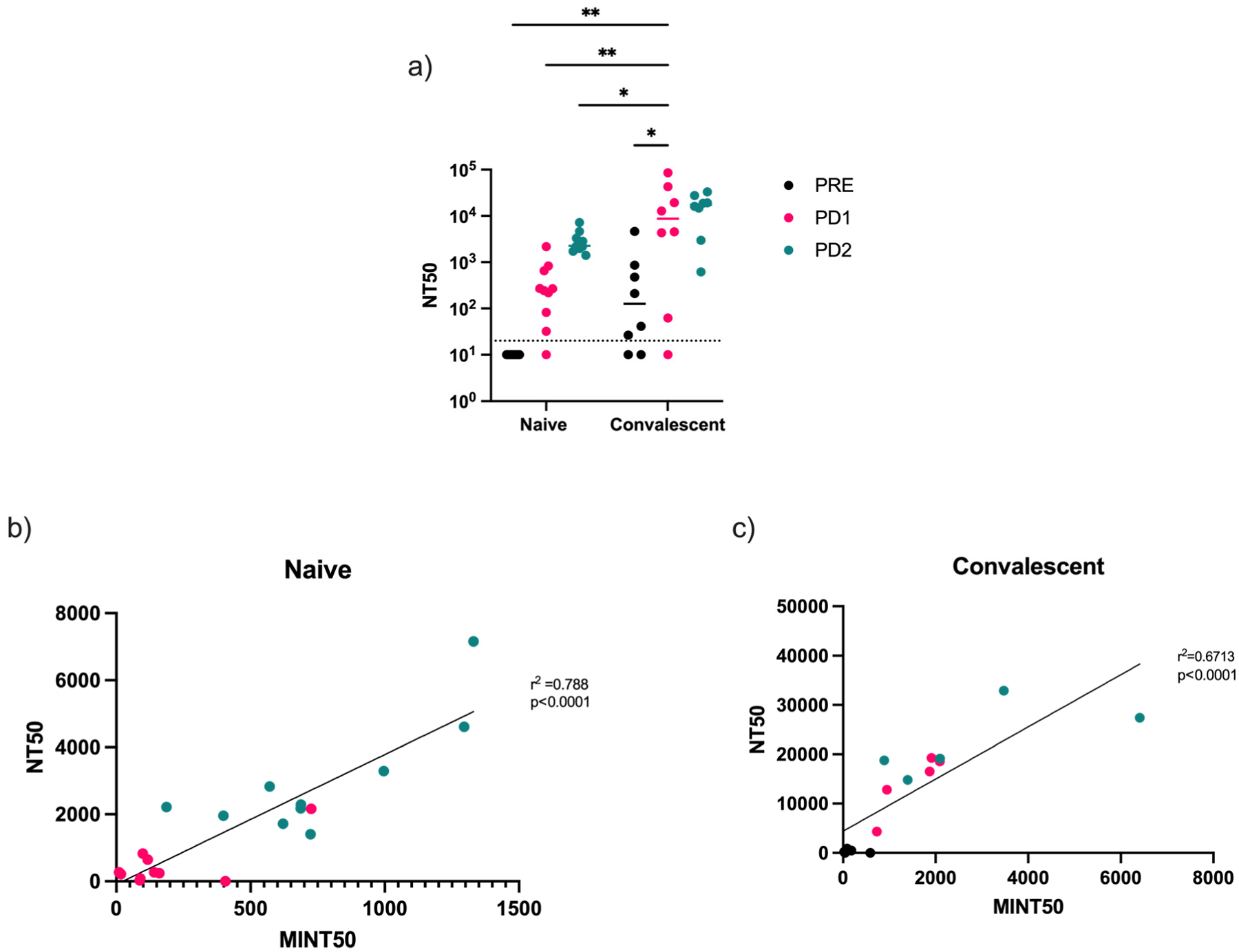
Virus neutralization was correlated with receptor-binding inhibition in SARS-CoV-2-naïve and -convalescent vaccinees. Virus neutralization was measured with a surrogate virus neutralization assay using a recombinant VSV pseudotyped with SARS-CoV-2 prototype variant spike protein further expressing GFP. (**a**) NT_50_ values (the dilution factor at which 50% neutralization was achieved) were compared for both groups and correlated with MINT_50_ values in (**b**) naïve and (**c**) convalescent samples (two-way ANOVA, * *p* < 0.05, ** *p* < 0.01).

To help establish the validity of the MINT assay, we compared our inhibition results to virus neutralization measured using an rVSV-based pseudotyped-virus neutralization assay (results reported as NT_50_). Similarly to receptor-binding inhibition, neutralization increased following vaccination in both the naïve and convalescent groups. Neutralization in the convalescent group was higher after dose 1 compared to two vaccine doses in the naïve group. To determine the potential for the MINT assay to predict virus neutralization, we correlated our results from the MINT assay with the results from the neutralization assay. In both the naïve and convalescent groups, MINT_50_ and NT_50_ values had strong correlations. (Figure 5b, r^2^ = 0.788; Figure 5c, r^2^ = 0.671). Additional work needs to be conducted, as it appears that the predictive value of the MINT assay is stronger in the naïve group compared to the convalescent group. This could be due to variations in the interval between infection and vaccination in the convalescent group or other factors directly linked to higher variability in immune response to natural infection.

### 3.5. PRNT_50_ Correlates with Receptor-Binding Inhibition in Non-Human Primates

**Figure 6.**
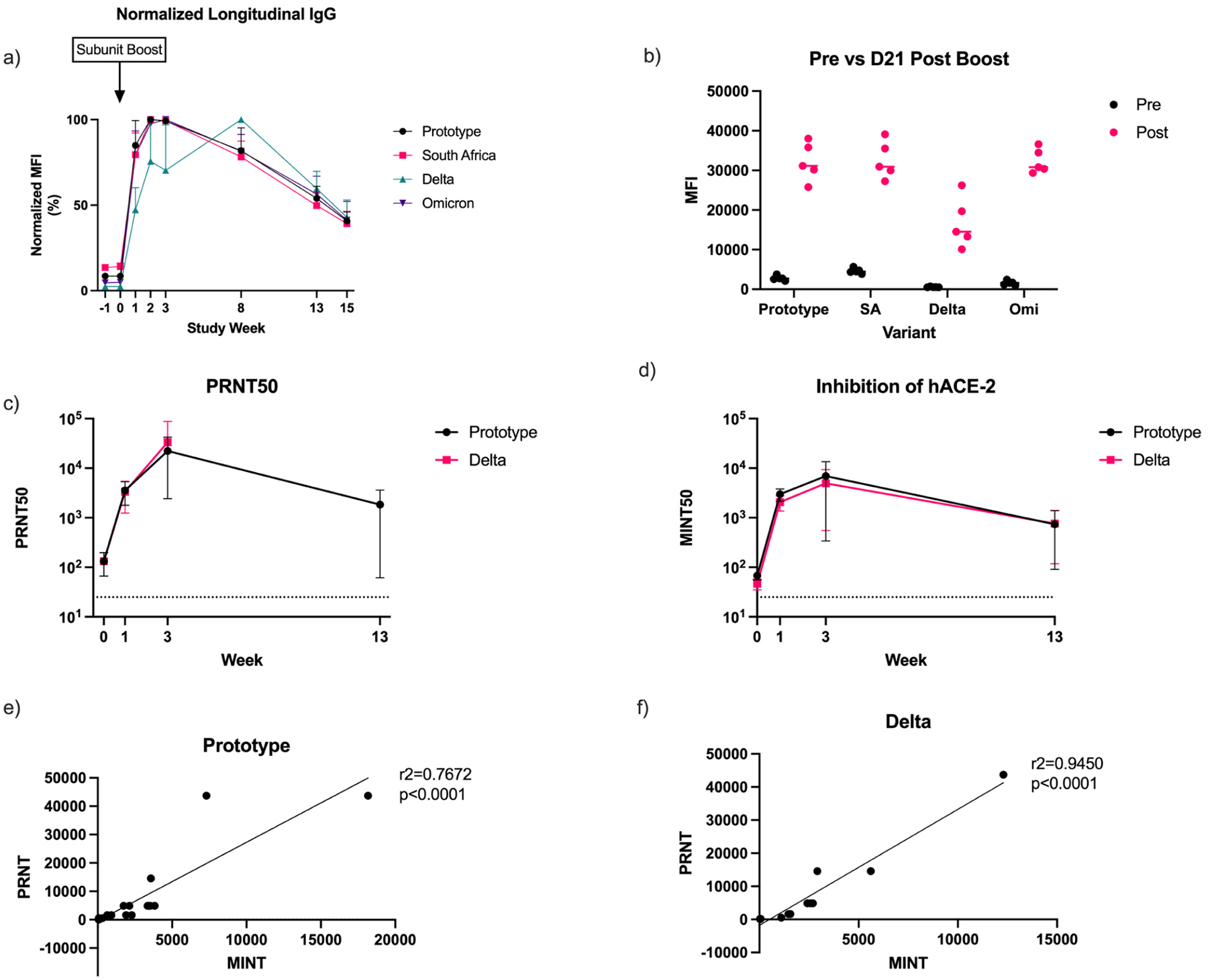
Multiplex Inhibition Test results correlate with the virus-neutralizing titers against the SARS-CoV-2 prototype and Delta variants observed in previously immunized NHPs after a heterologous protein vaccine booster, inducing broad cross-variant immunity. (**a**) Serum IgG titers against the prototype, South Africa, Delta, and Omicron variant spike proteins post-booster. The MFIs were normalized to the mean for each variant. (**b**) The serum IgG titers against spike protein were compared prior to vaccination and 21 days post-vaccination for each variant. (**c**) Serum PRNT_50_ titers against the wildtype prototype and Delta variant isolates prior to the booster and 1 week, 3 weeks, and 13 weeks post-booster. (**d**) MINT_50_ Titers against prototype and Delta variant SARS-CoV-2 prior to the booster and 1 week, 3 weeks, and 13 weeks post-booster. Correlation between MINT_50_ and PRNT_50_ values for the (**e**) prototype and (**f**) Delta SARS-CoV-2 variants.

To further show the utility of the inhibition assay, we compared MINT data to PRNT data for additional SARS-CoV-2 variants. Given the speed at which new variants of SARS-CoV-2 have emerged, it is important to establish a test that can rapidly measure the neutralization of new variants. In this study, non-human primates were originally vaccinated with two doses of an adenovirally vectored SARS-CoV-2 vaccine (prototype antigen) and received a recombinant protein subunit vaccine booster ~7.5 months later (prototype antigen). We first measured the post-booster anti-spike IgG for the prototype, South Africa, Delta, and Omicron variants (Figure 6a,b). IgG antibodies against multiple variants rapidly increased following booster administration. The PRNT data show the rapid induction of homologous and heterologous neutralizing titers as soon as 1 week post-booster. Neutralizing titers continued to increase up to 3 weeks post-booster and remained elevated even 13 weeks later at a level higher than prior to the booster vaccination (Figure 6c). Inhibition titers determined with the MINT assay also reflect the trend in neutralizing titers. We see rapid increases by week 1 and elevated inhibition titers through week 13 (Figure 6d). Additionally, there are strong correlations between the PRNT_50_ and MINT_50_ titers for both the prototype (Figure 6e, r^2^ = 0.77) and Delta (Figure 6f, r^2^ = 0.95) SARS-CoV-2 variants. Both the inhibition assay and neutralization assays confirm that a booster with the subunit vaccine rapidly recalls the immune response to multiple SARS-CoV-2 variants that was established by the prototype adenovirally vectored vaccines. Overall, these results demonstrate the potential capabilities of the MINT assay to detect broadly neutralizing responses. Additional work is needed to demonstrate whether the MINT assay can be effectively used for additional variants, as well as for other viruses, but the current results suggest that the MINT assay can be used as a tool to predict virus-neutralizing titers in situations where traditional neutralizing assays may not be available or difficult to establish.

## 4. Discussion

The goal of this study was to evaluate changes in antibody responses following vaccination in naïve and convalescent individuals using a custom MIA platform in different assay configurations. Specifically, we investigated multiple aspects of this platform’s utility to assess vaccine-induced immunity, including antibody concentration, antibody avidity, and the inhibition of receptor binding as a surrogate for virus neutralization.

As expected in the naïve group, we observed increases in Ag-specific antibody responses following one and two doses of the vaccine. In the convalescent group, serum IgG levels increased following a single vaccine dose. In these individuals, antibody responses did not change significantly following the second vaccination. Our results indicate that natural infection may result in higher antigen-specific antibody concentrations compared to a single mRNA vaccine dose. In addition, when comparing the naïve and convalescent groups with two instances of antigen exposure, our results also indicate that natural infection and a single vaccine dose may result in higher antibody concentrations compared to receiving two vaccine doses without prior infection. These results support prior studies that found that infection followed by a single vaccination generated a higher avidity response against the Omicron variant compared to vaccination alone [24,34]. However, whether this is due to differences in the characteristics of the immune response to natural infection and vaccination or whether the duration between first and second antigen exposure is responsible needs to be investigated further.

Interestingly, our assay results also identified two individuals with questionable SARS-CoV-2 exposure status. The samples from these persons had pre-vaccination IgG concentrations that fell below the limit of detection of the assay. In addition, following vaccination, the antibody response of those two samples was analogous to SARS-CoV-2-naïve individuals rather than other convalescent individuals. PCR testing has been shown to have low rates of false-positive results [35,36], so while it is possible that these individuals had false-positive results, there may be other interpretations of the results. The results from these “convalescent” individuals suggest that a PCR-positive test may not necessarily indicate a sufficient amount of viral antigen being produced, resulting in a robust immune response to infection. It is possible that these individuals may have had a mild infection and simply did not develop a detectable immune response. Alternatively, these individuals may have also developed a weak immune response that waned overtime. The pre-vaccination samples from these individuals were collected 199 and 258 days post-symptom onset. Prior studies have suggested that there is variable waning in antibody titers and neutralizing antibodies that may be dependent on peak titers following infection [37–39]. Unfortunately, we do not have acute serum samples following infection and are unable to measure peak antibody titers to determine if this might be the case, but the post-vaccine response suggests that there was no prior immunity. Overall, these results provide evidence of the sensitivity of this assay platform, as we were able to identify individuals who were immunologically naïve despite reporting PCR-positive test results but who followed the post-vaccination patterns of naïve individuals.

In addition, our results indicate a bias of the immune response towards the original SARS-CoV-2 variant following vaccination with the first-generation mRNA vaccines. In naïve individuals, there is an increase in antibody reactivity towards the prototype variant following each vaccine dose. However, the relative antibody cross-reactivity towards other variants decreases following dose 2 in the naïve group, suggesting an increase in specificity towards the prototype spike protein. In the convalescent group, antibody reactivity against all variants increases following a single vaccine dose, but like the naïve group, reactivity to other variants decreases following the second dose. As previously mentioned, individuals in the convalescent group most likely were infected with the original prototype variant of SARS-CoV-2 or a closely related isolate. This could explain why, overall, there is a relatively narrow pattern of cross-reactivity in both groups. However, there appear to be differences in the formation of cross-reactive antibodies that may be driven by differences in antigen exposure during the first two immunizing events between the naïve and convalescent groups. The naïve group had received two vaccine doses, whereas participants from the convalescent group had only received a single vaccine dose at this time. It is unclear why this might have occurred, but it is possible that more limited antigen exposure from the mRNA vaccine does not generate as robust or diverse of an immune response as exposure to recombinant protein or whole virus. The timing between both antigen exposures may also play an important role. In the naïve group, there appears to be immune bias towards the prototype variant following vaccination, while natural infection followed by vaccination, as seen in the convalescent group, may help to increase the breadth of the immune response induced by vaccination alone. Others have also made similar observations, supporting our findings, that mRNA vaccination only may not induce as robust of a cross-reactive immune response compared to hybrid immunity (immunization + infection) [40]. Additionally, this idea of a biased immune response is not novel. Original antigenic sin, or immune imprinting, is the idea that the original exposure to an antigen plays a major role in subsequent responses to a similar antigen. This phenomenon is commonly associated with influenza [41,42], but there is evidence that supports immune imprinting for other pathogens, including SARS-CoV-2. Studies have shown that a bivalent mRNA booster is no more effective in generating cross-protective immunity than a booster with the original mRNA vaccine [43]. Another study found that individuals with prior infection with an early SARS-CoV-2 variant and subsequent reinfection with Omicron had lower anamnestic responses compared to previously naïve individuals who had primary Omicron infection [44]. This supports the idea of immune imprinting and may explain why, in our study, the naïve group experienced an increase in prototype-specific antibodies but a decrease in variant-specific antibodies following the second vaccine dose. The results from the convalescent group differ in that infection followed by vaccination increases antibodies with more specificity for all variants, again suggesting that hybrid immunity elicited by two different forms of antigen exposure can generate a more robust immune response than vaccination with two doses of the same vaccine. With the number of individuals receiving multiple different vaccines and experiencing infections from multiple variants, the mechanisms behind hybrid immunity to SARS-CoV-2 grow more complex. One study found that Omicron breakthrough infections are less immunogenic than breakthrough infections with Delta, and in either case, cross-neutralizing immunity was limited [45]. However, other studies suggest the opposite: that breakthrough Delta and Omicron infections following vaccination generates a robust, cross-reactive humoral immune response [46,47]. Clearly, more research needs to be conducted on hybrid immunity and how different antigenic combinations contribute to a protective immune response.

We also measured receptor-binding inhibition using a multiplex inhibition test. In the naïve group, there was a clear increase in inhibition following each vaccine dose. There was a similar trend in the convalescent group. However, the increase following dose 1 was far greater than that observed after dose 2. Interestingly, the convalescent pre-vaccination group had nearly undetectable MINT_50_ titers. However, those results appear similar when compared to MINT_50_ titers for the naïve group post-dose 1. To help establish the validity of the MINT assay to predict functional responses, we compared our inhibition results to virus neutralization. In both the naïve and convalescent groups, MINT_50_ and NT_50_ values showed strong correlations. The correlation was stronger in the convalescent group; however, whether this was simply due to generally higher neutralizing titers or whether this suggests that prior infection status may play a role in the ability of the MINT assay to predict virus neutralization needs to be determined.

To further demonstrate the capabilities of the MINT assay, we also correlated MINT_50_ titers to PRNT_50_ titers for multiple SARS-CoV-2 variants in a separate NHP vaccination study. Previously, adenovirally vaccinated NHPs were boosted with an adjuvanted SARS-CoV-2 spike protein vaccine. The animals showed rapid increases in both neutralizing and inhibition titers that persisted up to 13 weeks post-booster. Additionally, the protein subunit booster contributed to the breadth of the immune response against heterologous variants despite also being based on the prototype variant sequence. This contrasts with what was observed in the human samples in which boosting with the mRNA vaccine biased the immune response towards the prototype variant. This is a potential differentiation between the mRNA and protein subunit vaccines that warrants future investigation. In addition, the MINT_50_ and PRNT_50_ titers had strong correlations for both the prototype and Delta SARS-CoV-2 variants, which suggests that the inhibition assay could function as a potential surrogate for virus neutralization in settings where the use of traditional neutralization assays is limited. The multiplex nature of the MINT assay provides a critical advantage over traditional ELISA-based inhibition tests in that it allows for simultaneous testing of multiple antigens in the same well.

Our attempts to establish a surrogate virus neutralization assay is not unique; however, the use of the MIA platform does represent a novel approach to those efforts [48]. Multiple studies have conducted ACE-2-binding inhibition assays using ELISA platforms [49–52]. While these studies found similar moderate to strong correlations between their inhibition assay and a cell-based neutralization assay, additional work needs to be conducted before inhibition assays can act as surrogate neutralization assays. Despite current limitations, these efforts may have important implications in the development of cell-free and virus-free surrogate assays that are much less resource-intensive and time-consuming when compared to traditional neutralization assays.

In conclusion, we were able to evaluate differences in antibody concentrations, antibody avidity, and antibody inhibition of SARS-CoV-2 spike and ACE2-binding between naïve and convalescent vaccinated populations using variations of assays based on our custom MIA platform. In naïve individuals who had no prior exposure to SARS-CoV-2, a three-week interval successfully boosted the immune responses; however, this was not the case in those with previous SARS-CoV-2 infection. Our data suggest that antibody responses may have reached a maximum level in convalescent individuals following a single vaccine dose. The second dose, three weeks after the first dose of mRNA vaccine, had minimal further effect on any of the antibody response parameters measured, though it potentially narrowed any cross-reactivity. This could suggest that the booster interval of three weeks may have been too short for a convalescent population to benefit significantly. This finding may be important when determining schedules for SARS-CoV-2 vaccines in the future. It also appears that multiple doses of the mRNA vaccine further increased avidity against the prototype antigen at the expense of other antigen variants. Interestingly, in a primate study where animals received an adenoviral vector vaccine followed by a subunit protein vaccine, more cross-reactivity was seen after the follow-up subunit vaccine. Whether this represents a difference in the underlying immune response to the different vaccine technologies is something which warrants further investigation as we optimize the use of different vaccine technologies for different indications. While this study focuses on assessing the antibody response to vaccination, it should be noted that cell-mediated immunity likely also plays an important role in vaccine efficacy. Expanding the MIA platform to analyze aspects of cell-mediated immunity represents another potential avenue for future research [53–56].

In the context of SARS-CoV-2, we have been constantly facing emerging variants of concern, highlighting the importance of developing a rapidly adaptable platform that will allow us to respond to new viral variants. This also has broader implications in that we can rapidly test vaccine responses to many different viral diseases that may emerge or re-emerge in the future. Additionally, the lowered biosafety risk for MIA assays also makes this a potentially useful tool for testing antibody reactivity in low-income and resource-restricted regions as we have previously shown [30,57]. Overall, we are still in the process of establishing a system to rapidly test multiple aspects of the antibody response to infection and vaccination that can further be adapted for the development of other vaccines.

## Data Availability

The data presented in this study are available on request from the corresponding author.
